# Inter and intradevice assessment of microperimetry testing in aging eyes

**DOI:** 10.1038/s41598-024-51539-0

**Published:** 2024-01-10

**Authors:** Leonard M. Coulibaly, Hamza Mohamed, Philipp Fuchs, Ursula Schmidt-Erfurth, Gregor S. Reiter

**Affiliations:** 1 Department of Ophthalmology and Optometry, Vienna Clinical Trial Centre (VTC), Medical University of Vienna, Währinger Gürtel 18-20, 1090 Vienna, Austria; 2 Laboratory for Ophthalmic Image Analysis, Department of Ophthalmology and Optometry, Medical University of Vienna, Vienna, Austria

**Keywords:** Ageing, Retinal diseases

## Abstract

Microperimetry (MP) is a psychometric examination combining retinal imaging and functional sensitivity testing with an increasing importance due to its potential use as clinical study outcome. We investigated the repeatability of pointwise retinal sensitivity (PWS) on the most advanced commercially available MP devices under their standard setting in a healthy aging population. Two successive MP examinations on both MP-3 (NIDEK CO., Ltd., Gamagori, Japan) and MAIA (CenterVue S.p.A. (iCare), Padova, Italy) were performed on healthy aging subjects in a randomized order. PWS repeatability was analysed for different macular regions and age groups using Bland-Altmann coefficients of repeatability (CoR). A total of 3600 stimuli from 20 healthy individuals with a mean age of 70 (11) years were included. Mean CoR in dB were ±4.61 for MAIA and ±4.55 for MP-3 examinations. A lower repeatability (p=0.005) was detected in the central millimetre on MAIA examinations. Higher subject age was associated with a lower repeatability of PWS on both devices (both p=0.003). Intra-device correlation was good (MAIA: 0.79 [0.76–0.81]; MP-3: 0.72 [0.68–0.76]) whereas a moderate mean inter-device correlation (0.6 [0.55–0.65]) could be detected. In conclusion, older subjects and the foveal region are associated with a worse pointwise repeatability.

## Introduction

Visual function can be assessed using different patient-involving testing methods. Best corrected visual acuity (BCVA) has been established as the simplest, most widely used quantitative psychometric parameter to measure visual function. Change of BCVA has been used as primary endpoint in landmark clinical trials on common retinal diseases^1–3^. Meanwhile BCVA only describes the broad functionality of the central macular area and offers limited insights for small and/or extrafoveal areas with retinal dysfunction. Regarding retinal disease with a specific location such as non-fovea involving geographic atrophy (GA) secondary to age-related macular degeneration (AMD) this limitation is evident^4^. Moreso, early retinal disease stages are often characterized by a subclinical manifestation with no significant influence on BCVA. Small but important pathologic changes might already be objectified using alternative functional testing that could provide crucial insight into future disease progression.

Microperimetry (MP, also called fundus controlled perimetry) has proven to be a robust psychometric method assessing point-wise retinal sensitivity (PWS) especially in patients with a reduced fixation ability, common in pathologies affecting the foveal region. Recent publications have highlighted its use for early detection of diabetic retinopathy (DR)^5^, AMD^6^ or Stargardt disease (STGD)^7^. By combining real-time eye tracking technology with scanning laser ophthalmoscopy and point-wise stimuli projection, MP offers a reliable testing method that enables a more comprehensive mapping of the complete macular functionality compared to BCVA or conventional perimetric testing alone in patients with retinal disease^8^. MP combines retinal imaging with functional testing highlighting potential structure-function correlations for healthy and pathologic retinas alike. MPs role as a novel functional clinical endpoint for future trials has been proposed for studies on acquired retinal disease like intermediate^9^ and late-stage non-neovascular AMD^4^, as well as for hereditary disease like STGD^10^ or retinitis pigmentosa^11^.

The most advanced commercially available and well-established MP devices are the Macular Integrity Assessment device (MAIA, CenterVue S.p.A. (iCare), Padova, Italy) and the MP-3 (NIDEK CO., Ltd., Gamagori, Japan). Although both devices have the same purpose, different technologic components are used by the manufacturers. While the MAIA uses a patented combination of a light-emitting diode (LED) and a steering mirror to project and position the light stimuli on live fundus images with a dynamic range of 36dB, the MP-3 uses a liquid crystal display (LCD) for stimuli projection with a dynamic range of 34dB^12^. Further, the standard background luminosity on each examination differs from device to device. A background luminosity of 31.4 asb (10 cd/m^2^), as used in standard MP3 retinal sensitivity assessment, corresponds to photopic vision which is mainly mediated by cones^13^. Meanwhile the standard background luminosity of MAIA exams is 4 asb (1.27 cd/m^2^) which corresponds to mesopic vision and is mediated by rods as well as cones^14^. Mesopic background illumination has been established as the most common test condition used in MP^12^.

Previous studies have evaluated test-retest repeatability of MAIA and MP3 devices in healthy patients^15–18^. These studies have mostly analysed only one of the two devices and often on healthy young subjects. Regarding the high interest in MP as functional endpoint in future clinical trials and its growing use in everyday clinical practice we propose a comprehensive, structured analysis on test-retest repeatability for MAIA and MP-3 examinations on the same day and under similar external (device-unrelated) conditions on healthy aged eyes. We believe that comparing the standard settings of each manufacturer is of relevance for the every-day clinical use of MP devices. Further we analyze the role of stimuli eccentricity and subject age on test-retest repeatability.

## Methods

This study adhered to the tenets of the declaration of Helsinki and was approved by an Institutional Review Board. All participants have given their written informed consent before any study procedure was performed.

### Study population

At least fifty-year-old individuals without diagnosed retinal pathologies on the study eye were recruited during routine visits in the outpatient clinic of the department of ophthalmology of the Medical University of Vienna. Presence of intermediate (≥ 63 µm diameter) or larger drusen, macular neovascularisation (MNV), retinal pigment epithelium atrophy, significant epiretinal membrane or any sign of haemorrhage in the study eye as well as previous anti VEGF-therapy were considered exclusion criteria. Further any media opacity, advanced cataract or any sign of glaucoma (c/d ratio > 0.7 or history of ocular high-pressure) in the study eye as well as any history of retinal vein occlusion in the fellow eye were considered exclusion criteria. Only the presence of small drusen also termed drupelets (< 63 µm) was regarded as unspecific physiologic symptom of aging and therefore tolerated^19^. Only one eye per patient was eligible for inclusion. If both eyes were eligible, the one with better OCT-imaging quality was selected before initiating the microperimetry examinations.

### Testing protocol

A spectral-domain optical coherence tomography (SD-OCT) volume scan consisting of 97 B-scans using a Heidelberg Spectralis HRA+OCT (Heidelberg Engineering, Heidelberg, Germany) was performed prior to the classification of a healthy retina and was reviewed by a retinal expert for any of the above-mentioned exclusion criteria. After having received mydriatic eye drops (0.5% Tropicamide) to guarantee an adequate pupil dilation, two successive MP examinations on both MP-3 (NIDEK CO., Ltd., Gamagori, Japan: MP3 I and MP3 II) and MAIA (MAIA, CenterVue S.p.A. (iCare), Padova, Italy: MAIA I and MAIA II) were performed on the study eye, amounting to a total of four examinations. The sequence in which the devices were acquired was randomized using a validated automated randomization-tool (https://www.randomizer.at/) with a block size of 4, to minimize any bias related to weariness or a learning effect. All examinations were performed by a single experienced examiner in a dark and quiet room without windows (< 1 lux) while an eye patch was placed on the fellow-eye. For both devices an identical automatic stimulation pattern and a 4-2 staircase strategy was selected. Stimuli size was set to Goldmann III for a duration of 200 ms and the first examination started with stimuli at 17dB. The follow-up function was used for the second examination on each device (MAIA II and MP3 II). Between each of the four examinations a mandatory, at least ten-minute break was held to prevent weariness. A total of three breaks were held over the course of a study visit (For example: 1st break between MAIA I and MAIA II; 2nd break between MAIA II and MP3 I and 3rd break between MP3 I and MP3 II). For both devices the standard testing mode was selected to guarantee repeatability in an every-day clinical setting.

**Figure 1:**
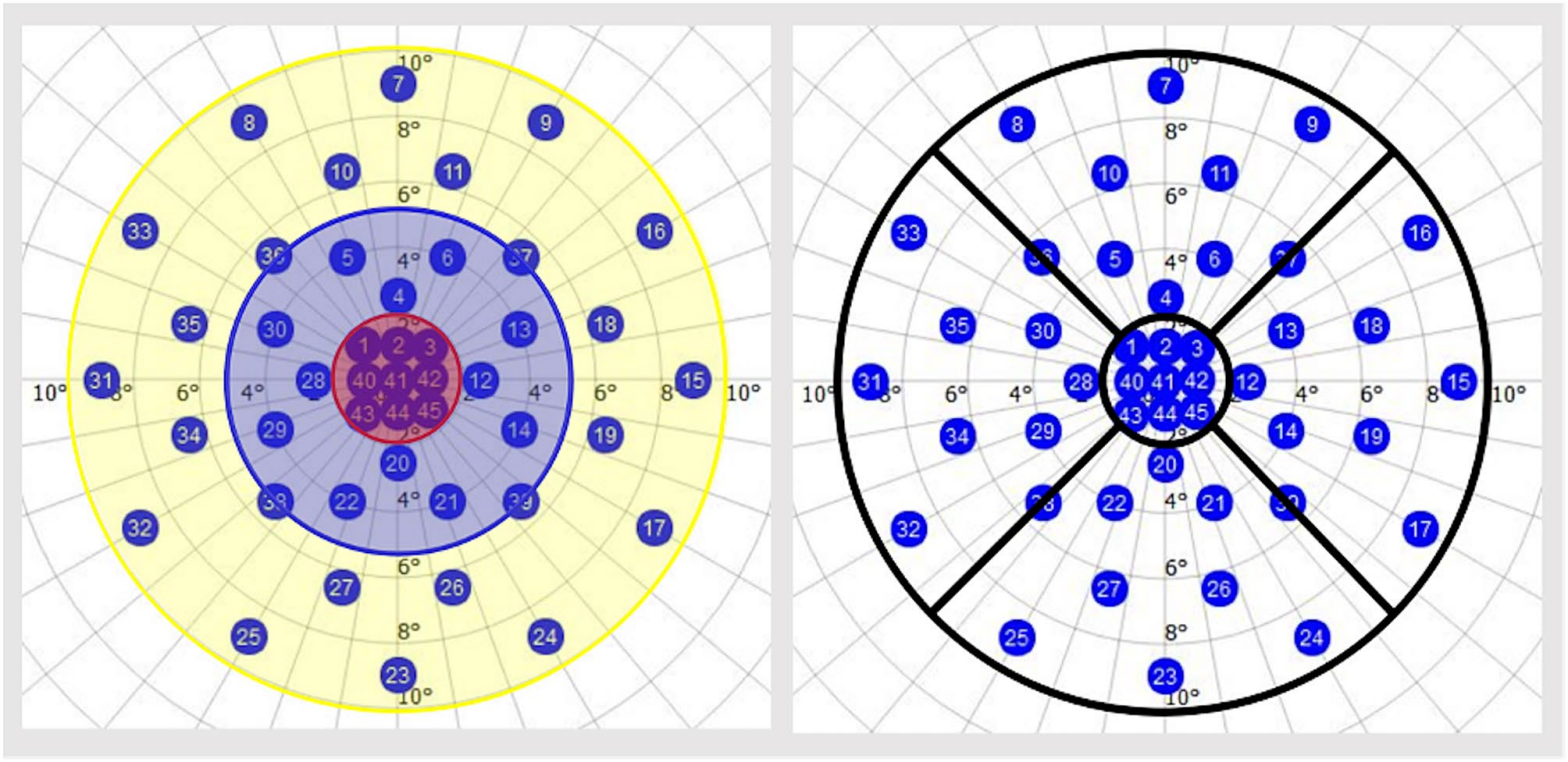
Stimuli pattern: foveal (red), parafoveal (blue) and perifoveal (yellow) region on the left; Superior, nasal, inferior and temporal region on the right.

The identical stimulation pattern for both devices was created using the *MP-3 Pattern-Editor* and consisted of forty-five stimuli points automatically centred at the fovea. Nine stimuli were within the foveal region (central mm), twelve stimuli were in the parafoveal region (1–3 mm) and twenty stimuli were in the perifoveal EDTRS region (3–6 mm). The superior, nasal, inferior and temporal region withheld eight stimuli each. Four stimuli points were not included in the subregion analysis as they were located on the border between subregions. The stimulation pattern for a right eye with designated macular subfields are represented in Fig. 1.

### Statistical analysis

Absolute PWS test-retest repeatability between consecutive microperimetry examinations was evaluated using coefficients of repeatability at a 95% level (CoR). These Bland-Altman coefficients of repeatability were calculated by multiplying the within-subject standard deviation by 1.96^20^. Percentages of agreement between the two consecutive measurements on the same device were calculated for absolute agreement (same dB value in both examinations) and within a ± 2dB range. Relative reliability between the two devices was assessed using interclass correlations (ICCs). ICC estimates and their 95% confidence intervals were based on a mean-rating (k = 2), 2-way mixed-effects model with absolute-agreement for examinations on the same device and consistency for examinations on different devices.

The role of stimuli eccentricity was assessed by analysing PWS repeatability in the foveal, the parafoveal, perifoveal region as well as in the inferior, superior, nasal and temporal quadrants (1–6 mm).

To assess the effect of aging on repeatability, participating individuals were divided into the three age groups: below 60 years, from 60 to 80 years and above 80 years. Hetero- or homogeneity of variances for the within-subject difference in our subgroup analysis were assessed using a Levene-test based on the median.

Fixation stability was quantified in relation to fixed circular regions centred at the fovea within a 2° and 4° diameter and categorized using the Fuji classification^21^. Therefore, a stable fixation was observed if 2° included greater than 75% fixation points, a relatively unstable fixation was observed if 2° included less than 75% and 4° includes more than 75% fixation points and an unstable fixation was observed if 4° included less than 75% of fixations points. Linear mixed models with device and consecutive measurement number as fixed factors were used to assesses differences in examination duration.

P-values <0.05 were considered statistically significant. All statistical calculations were performed using SPSS statistical package version 23 (SPSS Inc, Chicago, IL).

### Ethics approval and consent to participate

Ethics approval was obtained from the ethical commission of the Medical University Vienna in 2021(EK1399.2021).

## Results

**Figure 2:**
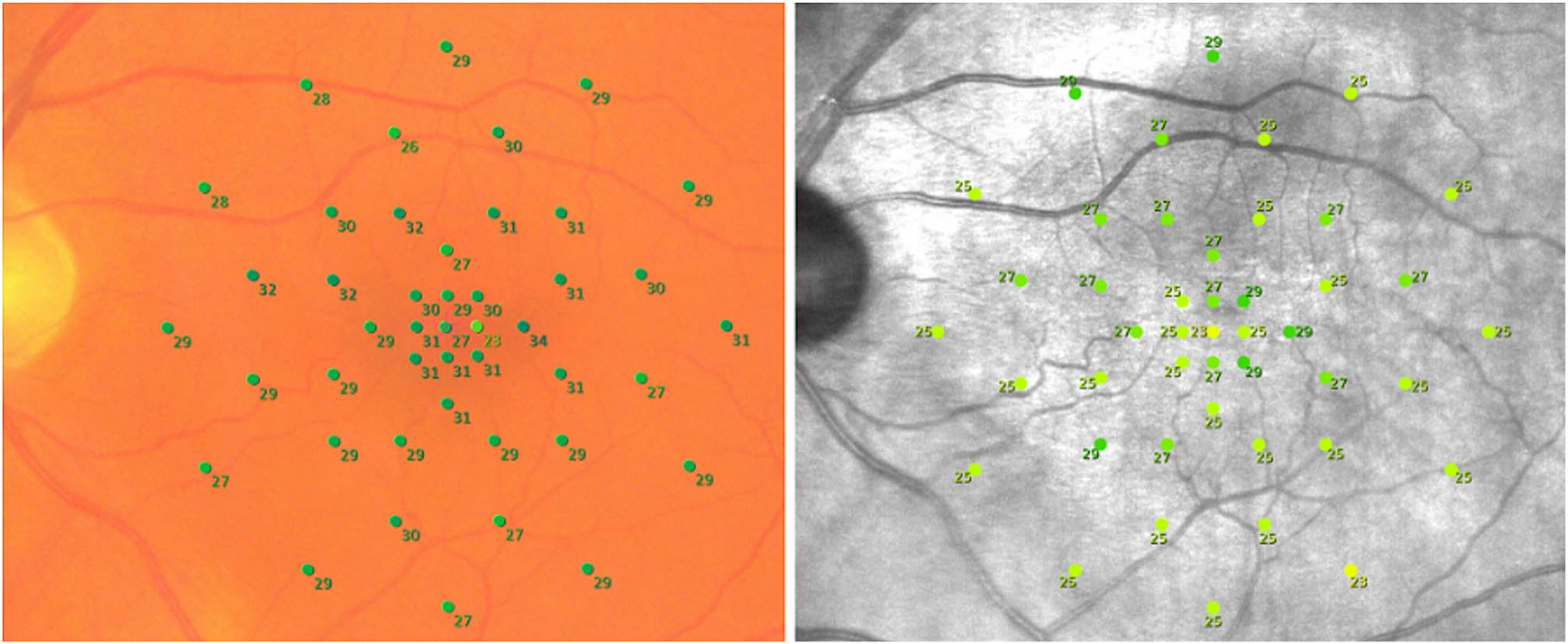
Results microperimetry examination for the same healthy subject with MAIA (right) and MP-3 (left). Stimuli points and measured pointwise retinal sensitivity in dB are marked in green.

Thousand-eight-hundred stimuli were acquired on each device, resulting in a total analysis of three-thousand-six-hundred stimuli from twenty eyes from twenty patients were included into the study. Figure 2 illustrates the examinations performed on each device. Five participants were under 60 and over 80 years old respectively. Ten participants were between 60 and 80 years old. Mean (SD) age of participating individuals was 69.75 (10.89). Overall mean retinal sensitivity (SD) was 25.44 (2.56) for MAIA and 28.87 (2.19) for MP-3. Divided over the macular regions, mean retinal sensitivity in the foveal region was 26.78 (2.49) and 29.58 (2.65), in the paracentral region it was 25.95 (2.43) and 29.54 (2.07) and in the pericentral region it was 24.54 (2.38) and 28.17 (1.89) for MAIA and MP-3, respectively.

### Test-retest repeatability

**Figure 3:**
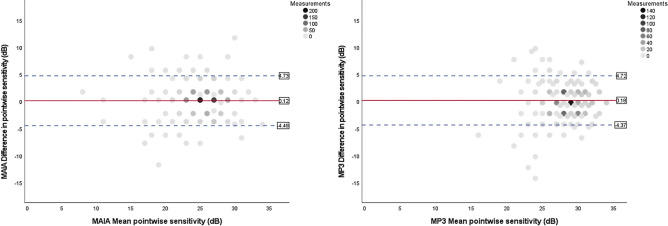
Bland–Altman plots of pointwise sensitivity (PWS) for and MAIA (left)and MP-3 (right) in healthy aging individuals. Horizontal dashed lines representing upper and lower limits of 95% of the mean (± 1.96 SD) and the horizontal red line representing the mean of the differences in PWS. Measurements refers to the number of stimuli summarised by one point on the Bland-Altman plot.

An absolute correlation between test loci for the two consecutive examinations on the same device were found in 50% for MAIA and 33% for MP-3. 83% and 85% of repeated stimuli were within a range of − 2 to + 2 dB for MAIA and MP-3, respectively. Bland-Altman Coefficients of repeatability were ± 4.61 dB for MAIA examinations and ± 4.55 dB for MP-3 examinations. The limits of agreement (LoA) according to the Bland-Altman plots were + 4.73 to − 4.49 for MAIA and + 4.72 to − 4.37 for MP3 (see Fig. 3).

Additionally, CoR were calculated in every macular subfield and for each age group on both devices. The results are summarized in Table 1.

**Table 1:** Repeatability for CenterVue MAIA and Nidek MP-3 in all subfields and for all age-groups.

Group	Nb of Stimuli	MAIA (dB)				MP-3 (dB)			
		SD Difference	CoR	Upper LoA	Lower LoA	SD Difference	CoR	Upper LoA	Lower LoA
Macula overall	900	2.35	$\pm$ 4.61	4.73	− 4.49	2.32	$\pm$ 4.55	4.72	− 4.37
Subfields									
Foveal	180	2.66	$\pm$ 5.21	5.16	− 5.28	2.36	$\pm$ 4.62	4.71	− 4.53
Parafoveal	240	2.19	$\pm$ 4.29	4.48	− 4.12	2.42	$\pm$ 4.74	4.04	− 5.44
Perifoveal	400	2.27	$\pm$ 4.45	4.54	− 4.36	2.1	$\pm$ 4.12	4.14	− 4.09
Superior quadrant	160	2.149	$\pm$ 4.21	4.34	− 4.08	2.2	$\pm$ 4.31	4.42	− 4.20
Nasal quadrant	160	2.002	$\pm$ 3.92	3.91	− 3.93	2.1	$\pm$ 4.12	4.05	− 4.19
Inferior quadrant	160	2.68	$\pm$ 5.25	5.50	− 5.00	2.17	$\pm$ 4.25	4.26	− 4.24
Temporal quadrant	160	2.08	$\pm$ 4.08	4.10	− 3.96	2.44	$\pm$ 4.78	4.64	− 4.92
Age-group in years									
Under 60	225	1.73	$\pm$ 3.39	3.56	− 3.22	1.91	$\pm$ 3.7	3.5	− 3.98
Between 60 and 80	450	2.37	$\pm$ 4.65	5.0	− 4.3	2.3	$\pm$ 4.51	4.76	− 4.26
Over 80	225	2.76	$\pm$ 5.40	5.02	− 5.79	2.66	$\pm$ 5.21	4.91	− 5.51

Over the complete macular region, no significant difference of repeatability could be detected between the two devices. Nonetheless, a Levene test based on the median detected significant differences for PWS repeatability regarding the eccentricity of the stimuli on examinations performed on the MAIA device with higher CoR within the foveal region compared to extrafoveal stimuli (p=0.005). While a similar high CoR was found for the inferior quadrant for MAIA, no significant difference of repeatability between extrafoveal quadrants was detected (p = 0.12). Further, homogenous variances were detected for the within-subject difference on repeated MP-3 measurements regarding eccentricity (p = 0.09) as well as among all extrafoveal macular quadrants (p = 0.81) indicating no difference in repeatability.

The Levene test revealed heterogeneity of variances regarding the different age groups (p = 0.003) indicating a repeatability decrease with increasing age. A significant difference was found between the age group “Under 60” and “Between 60 and 80” for MAIA (p=0.002) but not for MP3 (p = 0.08). Whereas a significant difference between the groups “Between 60 and 80” and “Over 80” could be detected in MP3 (p = 0.035) but not in MAIA examinations (p = 0.5). Significant differences were detected for both devices (MAIA p = 0.002; MP3 p = 0.001) by comparing the groups “Under 60” and “Over 80”.

As the device order was randomized before the first examination, half the participants started examinations on a MAIA device whereas the other half started with a MP-3 device. Repeatability of PWS relating to performed device order, independent from the manufacturer were almost identical with CoR of ± 4.45 and ± 4.78 for first and second device respectively.

### Inter- and intra-device correlation

ICCs [95%CI] between each of the devices are summarized in Table 2. Significant correlations (all p < 0.05) could be found between every measurement. While the mean intra-device correlation can be considered good, only a moderate inter-device correlation could be detected.

**Table 2:** Interclass correlation coefficients between each examination.

	MAIA I	MAIA II	MP-3 I	MP-3 II
MAIA I	1	0.79 [0.76–0.81]	0.62 [0.57–0.67]	0.59 [0.53–0.64]
MAIA II	0.79 [0.76–0.81]	1	0.59 [0.53–0.64]	0.59 [0.54–0.64]
MP-3 I	0.62 [0.57–0.67]	0.59 [0.53–0.64]	1	0.72 [0.68–0.76]
MP-3 II	0.59 [0.53–0.64]	0.59 [0.54–0.64]	0.72 [0.68-0.76]	1

**Figure 4:**
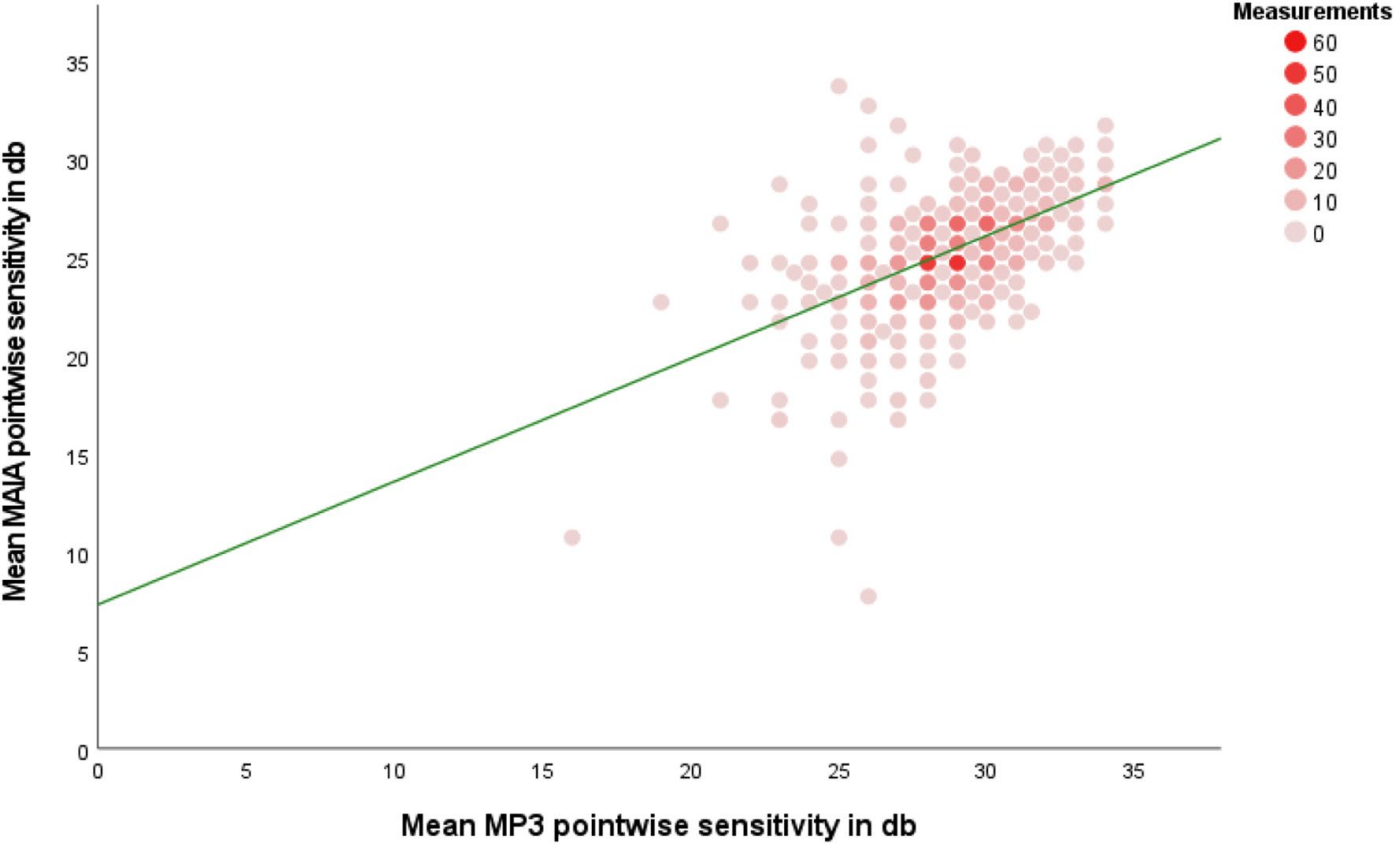
Scatterplot for mean MAIA and MP-3 point-wise retinal sensitivity measurements with the linear regression curve in green.

The linear regression equation between the mean of the two devices was:

$$Mean \,MAIA (dB)=0.63*\left(Mean MP3 \left(dB\right)\right)+7.34$$

with a moderate coefficient of determination (r^2^ at 0.29). A histogram analysis of the residuals confirmed a normal distribution. Figure 4 shows the graphic representation of the linear regression model between mean PWS results for MP-3 and MAIA examinations.

**Figure 5:**
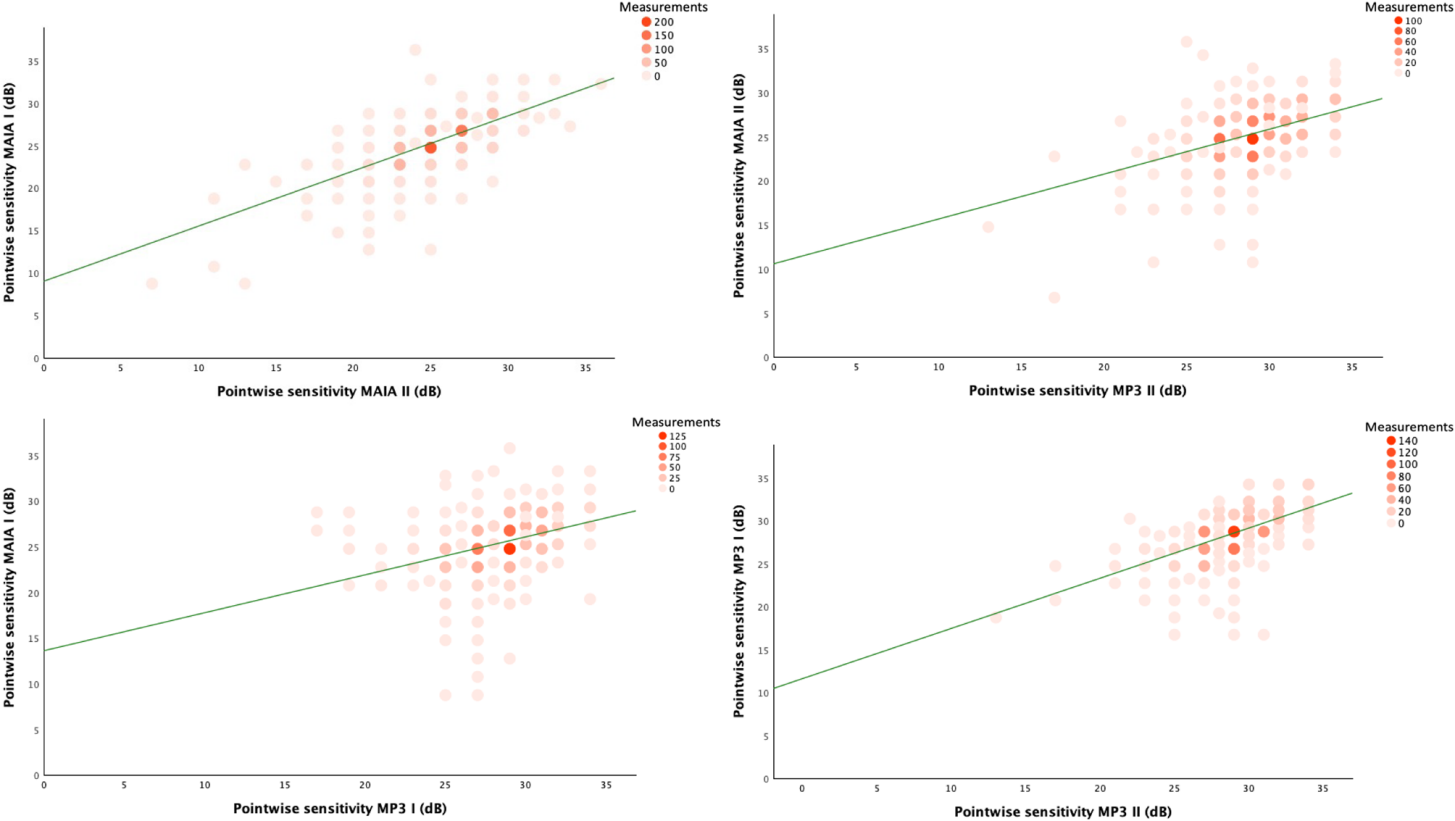
Scatterplot matrix for all performed examinations with linear regression curves in green.

Significant better coefficients of determination were found in a regression analysis within the same devices (MAIA I/MAIA II (r^2^=0.423) and MP-3 I/MP-3 II (r^2^=0.317)) compared between both devices (MAIA I/MP-3 I (r^2^=0.207) and MAIA II/MP-3 II (r^2^=0.182)). Graphical representations of intra-device correlations are summarized under Fig. 5.

### Examination duration

While the mean test duration for MAIA examination was 370 (42) seconds, it was 361 (75) seconds for MP-3. The first examination took a mean of 22 and 60 seconds longer (p < 0.001) compared to the follow up examination for MAIA and MP-3, respectively. A Pearson correlation detected a significant (p = 0.005) although low positive correlation (r = 0.309; N = 80) between subject age and examination duration.

### Fixation stability

Mean fixation was stable for both devices. No significant difference regarding fixation stability in the central 2° and 4° between consecutive testing on the same device, as well as between the two devices could be detected (Table 3).

**Table 3:** Mean fixation stability in central 2° and 4° for acquisition order and for each device.

	Central 2° in % (SD)	P-value	Central 4° in % (SD)	P-value
Mean 1st Examination all devices	81.7 (18)	0.161	93.5 (9)	0.177
Mean 2nd Examination all devices	83.9 (18)		94.7 (7.7)	
Mean all MAIA Examinations	83.9 (17.7)	0.108	94.6 (7.2)	0.298
Mean all MP-3 Examinations	81.1 (18.7)		93.7 (9.4)	

## Discussion

While test-retest repeatability for Nidek MP-3 and CenterVue MAIA, as well as an inter-device comparison in healthy subjects have been published previously, this was often done on separate and mostly young (<50 years) study cohorts. To our knowledge we present the first comprehensive intra-device repeatability and inter-device comparison for MP-3 and MAIA on the same healthy aging individuals in a randomized setting. This study provides reference values for a healthy aging population for the most common commercially available MP devices using their standard setting. It will strengthen the reliability of MP examinations as study endpoint for future clinical trials and will help to correctly interpret follow up examinations in everyday clinical use.

Overall test-retest repeatability was satisfactory in both MAIA and MP-3 for healthy aging individuals considering that more than 80% of repeated measurements were within a ±2 dB range. A potential explanation for the higher absolute correlation in MAIA testing compared to MP3 (50% vs. 33%) might lie in the differing stimuli projection. From a technical point of view LED might offer a more precise and comprehensive stimuli projection on a specific retinal location compared to LCD projection where important information might become distorted during the projection process.

As expected, without presence of scotoma in the healthy individuals, no floor effect could be detected. Meanwhile, the highest luminance threshold of 36dB for MAIA was reached in >1% of cases, whereas the upper limit of 34dB for MP-3 was reached in 4.6% of measured stimuli. This underlines the previously stated hypothesis that MP-3 testing might exceed retinal sensitivity in healthy patients^16^.

The foveal area was associated with a significantly worse repeatability compared to extrafoveal areas on examinations performed with the MAIA. This might be attributed to a reduced rod density within the fovea^22^ leading to a worse repeatability under mesopic conditions. Furthermore, an analysis of background luminosity in perimetric testing suggested that mesopic testing conditions might be prone for insensitivities in identifying functional deficits due to a “redundancy” of target detection^23^. Interestingly, although non significative, the lowest CoR was found in the nasal quadrant on both devices, which has reportedly the highest cone density outside the fovea^22^.

Potential minimal deviations in image acquisition and subsequent stimuli positioning using the follow up function could lead to an assessment of retinal sensitivity in a slightly different location. This could be an additional explanation for the observed weaker repeatability within the central mm, as our testing grid concentrated many stimuli within a comparable small area. A higher repeatability is achieved outside the central 1 mm as the spacing between stimuli points increased.

Our presented findings match the reported mean retinal pointwise sensitivity measurements as well as test-retest repeatability of previous studies for both devices. Palkovits et al. analysed test-retest repeatability for Nidek MP-3 on ten healthy subjects and found similar mean PWS (29.8 ± 0.9), limits of agreement of the Bland-Altman plots (+ 3.54 and − 3.02 dB) and CoR (±3.3 dB)^16^. A similar reproducibility could be attained for test re-test repeatability with the CentreVue MAIA under mesopic conditions while comparing our results to those of Pfau et al. who found a CoR of $\pm$ 4.75 dB and similar LoA (+ 4.29 to − 5.08 dB)^24^ or Higgins, Bethany E. et al. who reported a SD of the within subject differences of 2.62 in a healthy population^15^.

While comparable, the repeatability coefficients found in our analysis were slightly higher than in most of the above-mentioned studies. While mean study participants age in most of the above-mentioned healthy study cohorts were comparably young (<50 years), only older patients (>50 years) were included in our analysis. Indeed, our results suggest that PWS repeatability decreases in older patients, giving a valid explanation for the slightly worse repeatability in our analysis. Regarding the higher age of patients suffering from retinal disease, this finding is invaluable for the planning and sample size calculation of future clinical trials.

The logarithmic decibel (dB) scale used to assess retinal sensitivity is not an absolute scale but related to the maximal stimulus intensity specific for each MP device^25^. In case of the MP3 and the MAIA the maximum luminosity is comparable, providing an almost identical decibel scale^18^. Theoretically PWS measured under identical conditions should be convertible from one device to the other^12^, although empirical analysis remained unsatisfactory^18,26^. An analysis by Balasubramanian et al. on 31 healthy subject using MAIA and MP-3 under identical conditions still revealed that a correction factor of additional 5.65 dB was needed for MP-3 examinations to reach no significant difference in PWS with MAIA examinations^18^.

Our reported intra-device correlation was good and while the inter-device comparison had a lower degree of correlation, it can still be considered moderate^27^. The differing background luminosity, potential discrepancy in stimuli characteristics (geometrical, temporal and spectral), due to different processed technical components are important drawbacks for an ideal comparison of the two devices explaining the observed weaker inter-device correlation. While comparisons between different devices and background luminosities have been performed^28^, we present the first comparison between MAIA mesopic and MP3 photopic testing which is in line with the standard real-world usage of both devices.

Repeatability between the first and the second used device was almost identical suggesting no influence of weariness or learning effect. Similar findings have been reported by other study groups^15^. The shorter test duration of the second examination should be attributed to the used “follow up” function offered by each device instead of a potential learning effect. While other studies highlighted the benefits of a training session, this could not be supported in our analysis. Although, previous experiences with MP testing were not inquired before inclusion into the study. Further we could demonstrate that there might be a positive association between older subject age and a longer test duration.

Mean fixation for both devices can be considered as “stable” using the Fuji classification^21^, therefore minor observed differences should be considered negligeable. Our results indicate comparable fixation stability between MAIA and MP-3 which is in line with results from other device comparison studies^29^.

The strengths of our study included the prospective design as well as the age group stratification, the randomization of performed device order and the comprehensive testing of repeated measurements on both MP3 and MAIA. The differing background luminosity as well as potential discrepancy in stimuli characteristics, due to different processed technical components prohibits ideal conditions for a perfect comparison of the two devices. It is noteworthy that the MP3 offers the option to simulate mesopic testing conditions. While a better correlation between devices may have been achieved using mesopic testing conditions on MP3, our aim was to explore the inter-device relation using the standard settings of both devices. We believe that our investigation even under differing conditions is worthwhile, considering the rising interest in microperimetry as additional functional testing method. Further, the PWS acquisition under standard settings might be more widespread and accessible in an every-day clinical setting. Therefore, the juxtaposition of the devices standard settings is a valuable addition for the contextualisation of MP-testing in a real-world setting. The number of participants might be considered a limitation, although it is consistent with previous similar studies, and we believe it to be sufficient to set a reference standard. Another weakness of our analysis is the single centre nature of the study, limiting its applicability for large multi-centre studies.

The observed remaining differences in repeatability between devices in this and preceding studies might be mitigated by correlating the measured retinal sensitivity to specific biomarkers within the retina. By matching sensitivity measurements with structural markers on optical coherence tomography, stimuli points might be attributed to the same exact area and a higher repeatability using different devices might be achieved. Future investigations will be needed to establish a robust structure-function correlation between loss of retinal sensitivity and disease specific imaging biomarkers.

In this analysis we could establish reference values PWS repeatability for the standard settings of MAIA as well as MP-3 examinations on the same healthy aging subject cohort. Older subjects age for both devices and the foveal region in MAIA mesopic testing are associated with a worse repeatability. Meanwhile caution must be applied while comparing sensitivity results from different devices as examinations between the devices standard settings cannot be considered interchangeable.

## Data Availability

The data that support the findings of this study are available from the corresponding author SE, upon reasonable request.
